# Hydroxypropyl-β-cyclodextrin for Delivery of Baicalin via Inclusion Complexation by Supercritical Fluid Encapsulation

**DOI:** 10.3390/molecules23051169

**Published:** 2018-05-14

**Authors:** Ying Li, Zhen-Dan He, Qian-En Zheng, Chengshen Hu, Wing-Fu Lai

**Affiliations:** School of Pharmaceutical Sciences, Health Science Center, Shenzhen University, Shenzhen 518060, China; li.ying@szu.edu.cn (Y.L.); hezhendan@szu.edu.cn (Z.-D.H.); 2016224035@email.szu.edu.cn (Q.-E.Z.); cs.hu@siat.ac.cn (C.H.)

**Keywords:** baicalin, hydroxypropyl-β-cyclodextrin, supercritical fluid, inclusion complexation

## Abstract

Over the years, various methods have been developed to enhance the solubility of insoluble drugs; however, most of these methods are time-consuming and labor intensive or involve the use of toxic materials. A method that can safely and effectively enhance the solubility of insoluble drugs is lacking. This study adopted baicalin as an insoluble drug model, and used hydroxypropyl-β-cyclodextrin for the delivery of baicalin via the inclusion complexation by supercritical fluid encapsulation. Different parameters for the complex preparation as well as the physicochemical properties of the complex have been investigated. Our results showed that when compared to the conventional solution mixing approach, supercritical fluid encapsulation enables a more precise control of the properties of the complex, and gives higher loading and encapsulation efficiency. It is anticipated that our reported method can be useful in enhancing the preparation efficiency of inclusion complexes, and can expand the application potential of insoluble herbal ingredients in treatment development and pharmaceutical formulation.

## 1. Introduction

**Figure 1:**
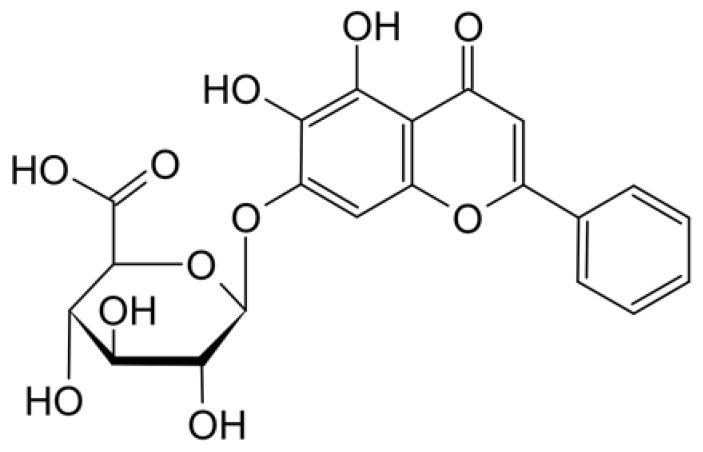
Structure of baicalin.

Baicalin is a flavonoid isolated from *Scutellaria*, a genus of flowering plants in the mint family *Lamiaceae*. Its chemical name is (2*S*,3*S*,4*S*,5*R*,6*S*)-6-(5,6-dihydroxy-4-oxo-2-phenyl-chromen-7-yl)oxy-3,4,5-trihydroxy-tetrahydropyran-2-carboxylic acid and has a molecular formula of C_21_H_18_O_11_ and molecular weight of 446.37. Baicalin possesses an *O*-diphenol hydroxyl (Figure 1), which is thought to be responsible for its reducibility. Baicalin has various biological activities including the antibacterial ability, anti-hypertension effect, diuretic ability, anti-inflammatory ability, detoxifying function, and anti-tumor effect. Previous research has found that baicalin protects the liver from liver injury, promotes fibroblast proliferation, protects human periodontal ligament fibroblasts, and inhibits the growth of parasites in vitro [1]. In clinical research, baicalin inhibits aldose reductase during the treatment of chronic complications of diabetes [2]. It also exhibits a positive effect on psoriasis patients with various skin lesions [2], and inhibits inflammatory edema of the retina [3]. With technological advances in the extraction of ingredients from herbal medicines, applications of baicalin in treatments of diseases such as cancers, AIDS, and cardiovascular diseases are worth further exploration [4].

Despite its clinical potential, pure baicalin has poor aqueous solubility, poor stability, and high susceptibility to oxidation and deterioration. These have jeopardized the clinical use of baicalin [5]. To increase the solubility and bioavailability of baicalin, a variety of dosage forms have been developed. For instance, baicalin has been formulated as a baicalin-PEG6000 dispersion or as phospholipid complexes for better dissolution [6,7]. By using stearic acid as a carrier, baicalin solid lipid nanoparticles (BC-SLNs) have also been prepared using a thin film ultrasonic method. In vitro studies have revealed that BC-SLNs have good drug release sustainability, with the bioavailability of baicalin being enhanced [8]. More recently, baicalin has been loaded into gelatin microspheres and transdermal formulations (e.g., water-soluble suppositories and sustained-release tablets) for better performance [9]. Despite these advances, currently most of these technologies for enhancing the solubility of baicalin are time-consuming and labor intensive or involve the use of toxic materials.

In this study, we adopted baicalin as a model of poorly soluble drugs, and used a cyclodextrin (CD)-based method mediated by supercritical fluid encapsulation to improve the solubility of baicalin. CD-based inclusion complexation can facilitate drug delivery by changing the molecular structure of poorly soluble drugs, by improving the physical and chemical properties of drugs, and by enhancing the affinity of drugs for gastrointestinal mucosa. Structurally, CD is a cyclic oligosaccharide consisting of a number of glucose molecules linked by α-1,4-glycosidic linkages. Its molecule resembles a truncated cone with a relatively hydrophobic cavity [10,11]. Hydrophobic molecules can, therefore, enter into the central cavity to form inclusion complexes. Owing to the hydrophilic exterior, the inclusion complexes can dissolve in water, thereby improving the stability and bioavailability of some water-insoluble substances [12]. Among the different CD carriers, one representative carrier is hydroxypropyl-β-CD (HP-β-CD), which is a derivate generated upon the substitution of the C2, C3, and C8 hydroxyl groups of β-CD. HP-β-CD possesses high water solubility, low renal toxicity, low hemolytic activity, and high safety. It was the first CD derivative applicable for injection preparations and has attracted extensive research interest [13]. Along with its ability to improve the volatility and instability of insoluble anticancer drugs and the active ingredients in traditional Chinese medicine, HP-β-CD has great application potential in future pharmaceutical formulation.

To generate inclusion complexes conventionally, a solution mixing approach is adopted. However, this method has various problems in the practical sense including high labor intensiveness, long operation time, the presence of residual solvents in products, low efficiency of inclusion complexation, and the difficulty in precisely controlling the physicochemical properties of the products [14]. Here, we propose solving these problems by using supercritical fluid encapsulation technology. As the use of organic solvents is not required during supercritical fluid encapsulation, the problem caused by solvent residues can be resolved [15,16,17,18,19]. In addition, the operation of supercritical fluid encapsulation is simple, and inclusion complexes can be generated with high efficiency in one step. By comparing the particle size, surface morphology, and other properties of the inclusion complexes generated by our method with those generated by the conventional one, our method has been shown to be more effective in mediating inclusion complexation and in controlling complex properties. With further development and optimization, our method has great potential in enhancing the bioavailability of poorly soluble drugs and to extend the use of those drugs in treatment development in the future. 

## 2. Results and Discussion

### 2.1. Structural Characterization of Inclusion Complexes

**Figure 2:**
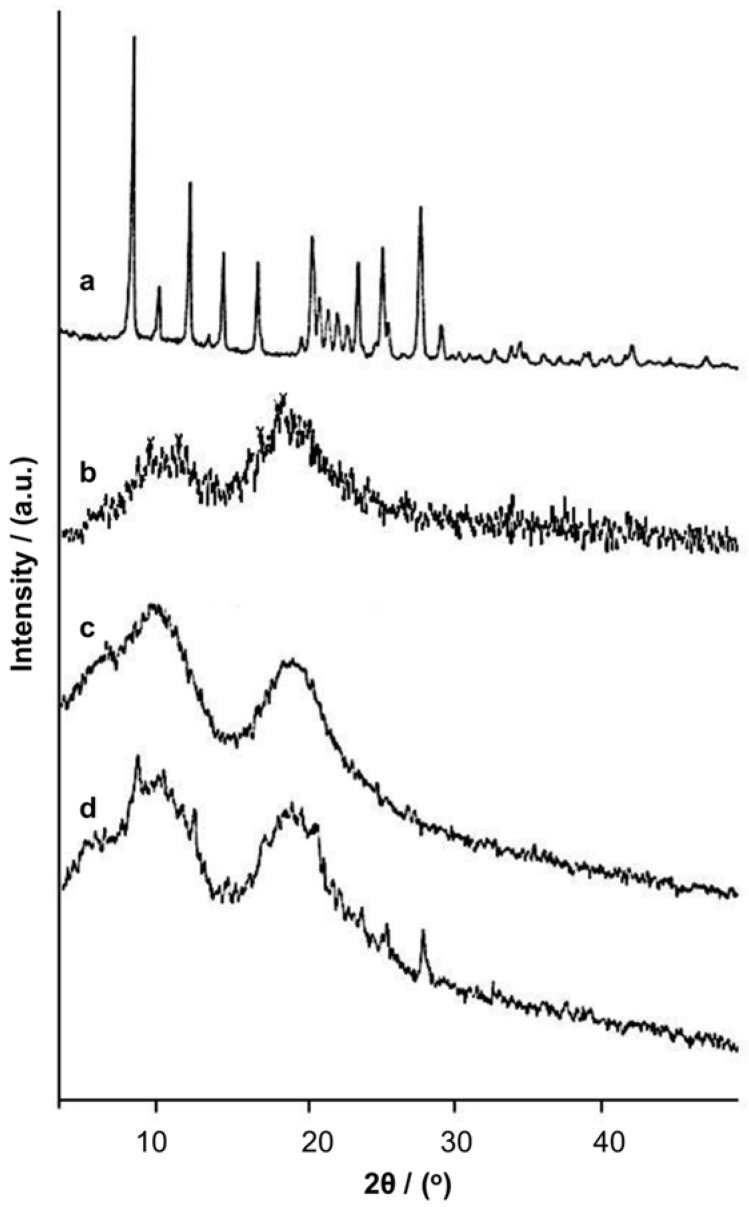
X-ray diffraction patterns of baicalin (a), HP-β-CD (b), the inclusion complexes prepared by conventional solution mixing (c), and the inclusion complexes prepared by supercritical fluid encapsulation (d).

Pure baicalin, HP-β-CD, the inclusion complexes prepared by conventional solution mixing, and the complexes prepared by the supercritical fluid technology were scanned by X-ray diffractometry (XRD). Characteristic diffraction peaks of baicalin were found at 8.5680, 10.3271, 12.3920, 14.6498, 16.9717, 20.6045, 21.1077, 23.7276, 25.4086, and 27.9602 (Figure 2). After the inclusion complexation, the diffraction peaks of the drug crystal became smaller, with the diffraction patterns of the inclusion complexes, either prepared by the conventional method or by supercritical fluid encapsulation, being very similar to that of HP-β-CD. This indicates that the drug is embedded into the cavity of HP-β-CD, which is characterized by the presence of a wide diffraction peak due to its amorphous structure.

**Figure 3:**
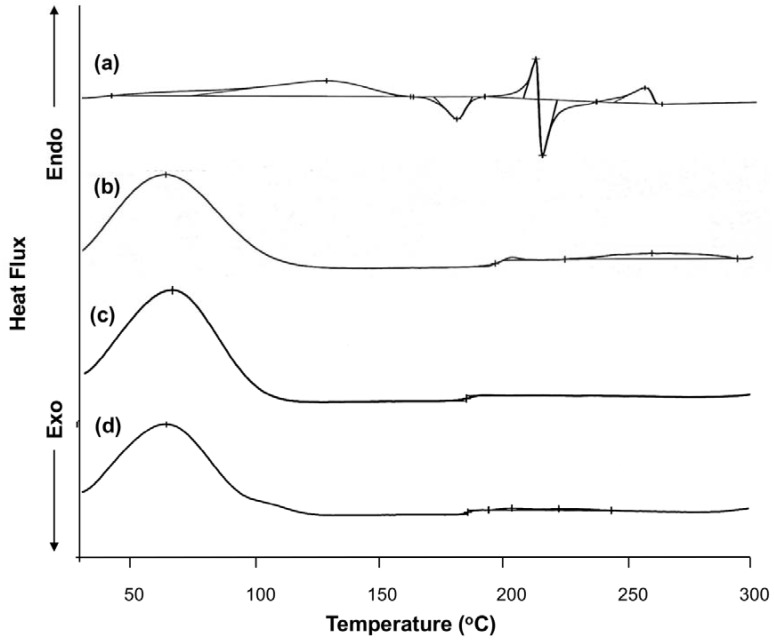
Differential scanning calorimetry thermograms of baicalin (a), HP-β-CD (b), the inclusion complexes prepared by conventional solution mixing (c), and the inclusion complexes prepared by supercritical fluid encapsulation (d).

The structures of the inclusion complexes generated from supercritical fluid encapsulation have also been examined by differential scanning calorimetry (DSC). Based on the results, pure baicalin was crystalline and had a relatively sharp endothermic peak near its melting point temperature range (Figure 3). It also had a wide absorption peak at 128.6 °C, which is the peak attributed to the dehydration of baicalin. The peak of 212.1 °C is the peak at which melting occurs. Compared to pure baicalin, the DSC thermograms of the inclusion complexes, prepared by either conventional solution mixing or supercritical fluid encapsulation, were similar to that of HP-β-CD. There was only a blunt endothermic peak near 65 °C, which can be attributed to the phase transition experienced by the complexes. The melting point peaks of baicalin disappeared, suggesting the success of the inclusion complexation through which the crystal characteristics of the drug are covered up. 

**Figure 4:**
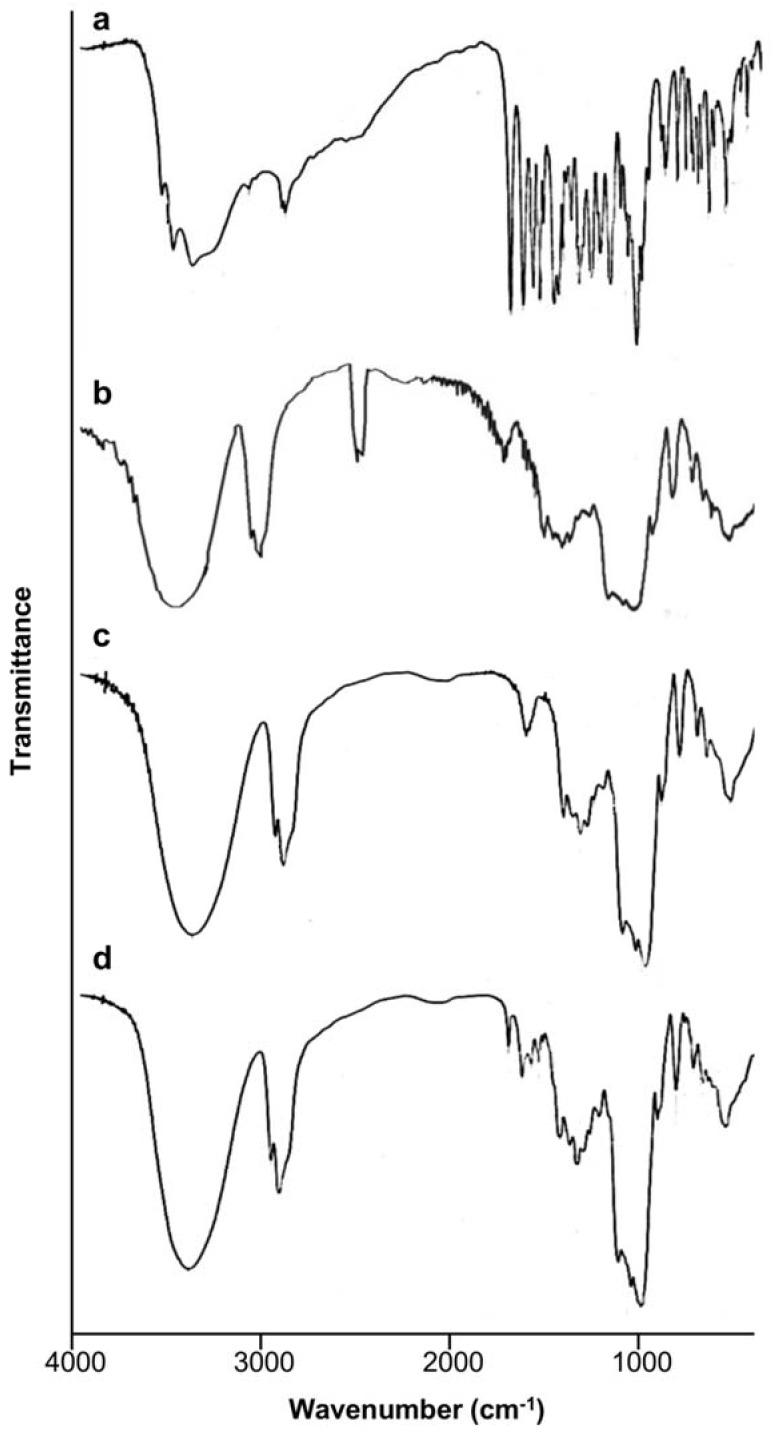
The infrared spectra of baicalin (a), HP-β-CD (b), the inclusion complexes prepared by conventional solution mixing (c), and the inclusion complexes prepared by supercritical fluid encapsulation (d).

To further confirm the structure of inclusion complexes, infrared (IR) spectrometry was employed (Figure 4). In the spectrum of baicalin, signals at 1612 cm^−1^ and 1665 cm^−1^ are characteristic peaks of carbonyl groups (1600–1700 cm^−1^). The presence of the double peaks ranging from 1500 cm^−1^ to 1700 cm^−1^ was caused by the characteristic peaks of C–H from benzene (1513 cm^−1^, 1556 cm^−1^). The peaks ranging from 1220 cm^−1^ to 1300 cm^−1^ were assigned to the characteristic peaks of C-H bending vibration and C=C expansion vibration (1211 cm^−1^, 1244 cm^−1^, 1317 cm^−1^, 1361 cm^−1^) of benzene. The peaks ranging from 1000 cm^−1^ to 1100 cm^−1^ were attributed to the vibration of benzene and to C–O–C expansion vibration (1000 cm^−1^, 1013 cm^−1^, 1094 cm^−1^, 1167 cm^−1^) [20]. All of these characteristic absorption peaks, however, disappeared in the IR spectra of the inclusion complexes prepared either by conventional solution mixing or supercritical fluid encapsulation. Instead, only the IR characteristic peaks of HP-β-CD (3405 cm^−1^ or 3397 cm^−1^, 2970 cm^−1^ and 2929 cm^−1^, 1655 cm^−1^, 1152 cm^−1^ or 1154 cm^−1^, 1085 cm^−1^, and 1036 cm^−1^) were present in the spectra of the inclusion complexes. This indicates that the drug molecules have completely entered into the cavity of HP-β-CD.

### 2.2. Surface Morphology of Inclusion Complexes

**Figure 5:**
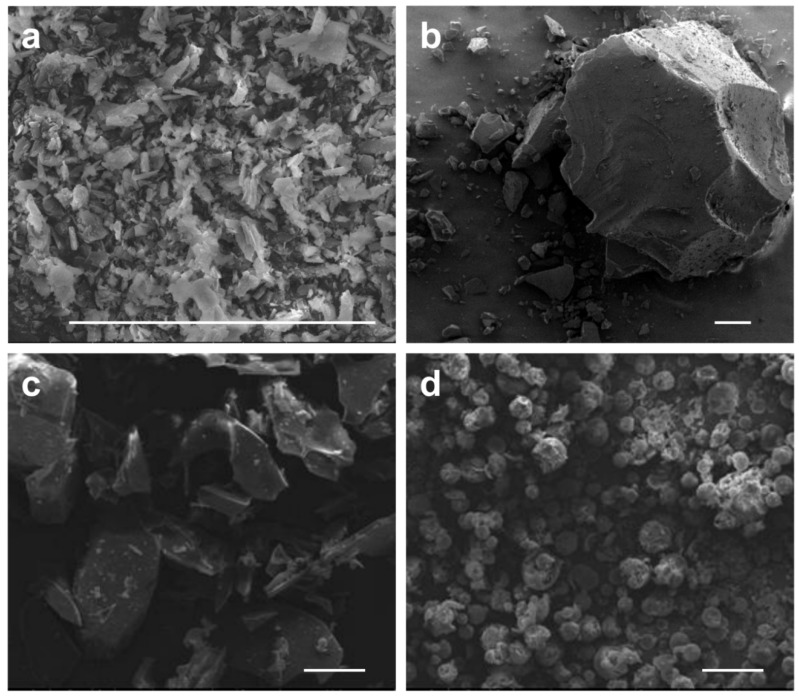
SEM images of baicalin (magnification = 1000×) (**a**), HP-β-CD (magnification = 200×) (**b**), the inclusion complexes prepared by conventional solution mixing (magnification = 200×) (**c**), and the inclusion complexes prepared by supercritical fluid encapsulation (magnification = 200×) (**d**).

The scanning electron microscopy (SEM) images of baicalin, HP-β-CD, and the inclusion complexes are shown in Figure 5. Baicalin presents a shorter flake and crystal morphology, which is similar to the morphology of crystalline drug substances. The surface morphology of the inclusion complexes prepared by conventional solution mixing was similar to that of HP-β-CD, existing in forms of lumps. However, the size and shape of the lumps were different. The morphology of the inclusion complexes prepared by supercritical fluid encapsulation presented a characteristic morphology of microspheres with relatively uniform grain size. Moreover, the particle size of the complexes was smaller than that prepared by the conventional method, and the crystal morphology of pure baicalin disappeared. This suggests that baicalin completely enters into the cavity of HP-β-CD, hence why the inclusion complexes mainly show the morphology of HP-β-CD.

### 2.3. Particle Size Analysis of Inclusion Complexes

**Figure 6:**
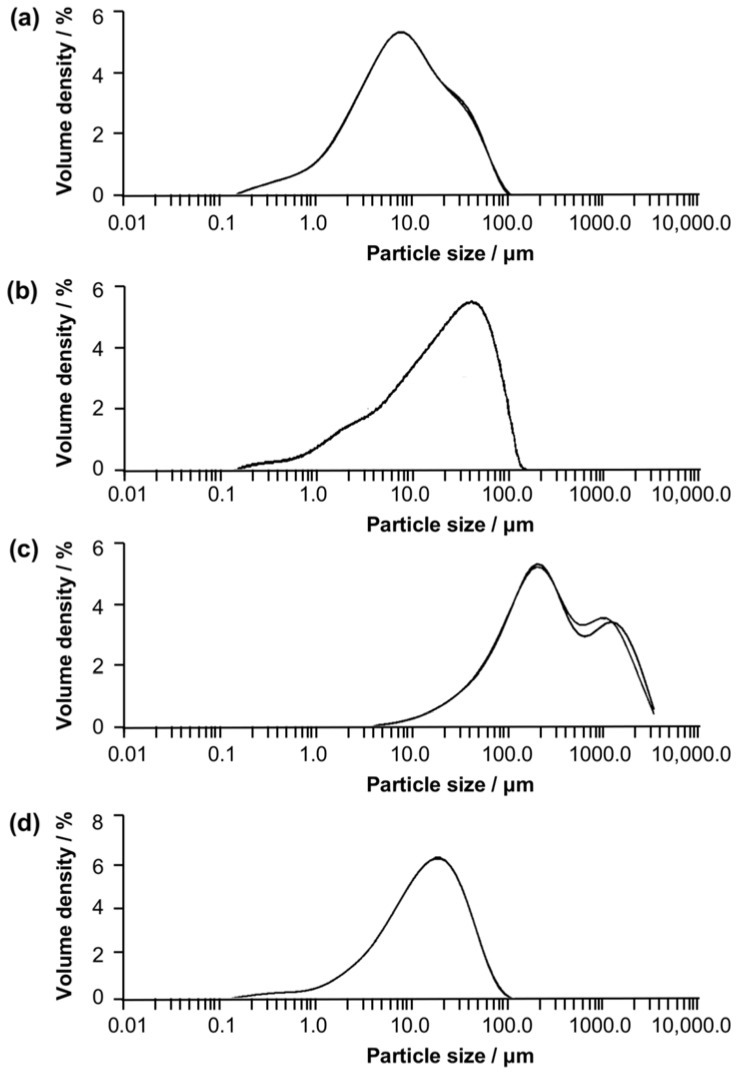
Particle size distribution profiles of baicalin (**a**), HP-β-CD (**b**), the inclusion complexes prepared by conventional solution mixing (**c**), and the inclusion complexes prepared by supercritical fluid encapsulation (**d**).

The particle size of pure baicalin showed a normal distribution uniformity, and was determined to be around 6.890 µm (Figure 6). The average particle size of HP-β-CD was 173.212 µm. The particle size of the inclusion complexes obtained by conventional solution mixing was significantly larger than that of those obtained by supercritical fluid encapsulation. Moreover, the particle size distribution of the conventionally prepared complexes was not uniform, showing a distribution of double peaks with the average particle size being 255.473 µm. In contrast, the particle size of complexes prepared by the supercritical fluid technology was significantly smaller, and displayed a homogeneous normal distribution with the average particle size being only 13.721 µm. The size was 19 times smaller than that of those prepared by conventional solution mixing and 13 times smaller than the particle size of HP-β-CD. These results are consistent with the observations made by SEM (Figure 5), and verified the merit of supercritical fluid encapsulation over conventional solution mixing in the preparation of inclusion complexes.

### 2.4. Modulation of Process Parameters 

After confirming the successful generation of inclusion complexes using supercritical fluid encapsulation, the impact of different process parameters (e.g., complexation time, temperature of the complexation chamber, pressure of the supercritical fluid, and the molar ratio of baicalin and HP-β-CD) on the properties of the complexes were studied for the optimization of the product quality. 

#### 2.4.1. Complexation Time

By keeping several process parameters constant (i.e., the molar ratio of baicalin and HP-β-CD = 1:2; the pressure of the supercritical fluid = 20 MPa; the temperature of the complexation chamber = 55 °C), the inclusion efficiency, drug loading efficiency, and water solubility of the inclusion complexes generated were determined after 3, 6, 10, and 14 h of inclusion complexation. Our results revealed that the water solubility of the generated complexes was positively related to the length of complexation time (Table 1). However, the impact of the length of complexation time on the inclusion efficiency and drug loading efficiency of the process did not seem to be significant. 

#### 2.4.2. Temperature of the Complexation Chamber 

By keeping several process parameters constant (i.e., the molar ratio of baicalin and HP-β-CD = 1:2; the pressure of supercritical fluid = 20 MPa; the complexation time = 8 h), the impact of the temperature of the complexation chamber on the inclusion efficiency, drug loading efficiency, and water solubility of the generated complexes were tested. In fact, the formation of inclusion complexes based on CDs was extremely sensitive to temperature and to competitive complexation with other small-molecule compounds (and their derivatives) found in biological systems (e.g., *n*-alkanes), although the reported impact of temperature on the stability of the generated complexes varies greatly among studies. While some studies have found that the formation of more stable complexes can be facilitated under lower temperature conditions [21,22,23], a high-temperature and high-pressure environment has also been reported in the literature as being able to facilitate the complexation process [24]. Such a discrepancy may have resulted from the variations in the mechanisms of different complexation processes. In this study, we found that an increase in temperature from 45 °C to 55 °C could improve the inclusion efficiency, drug loading efficiency, and water solubility of the generated complexes (Table 2). This may be because the preparation of CD-based inclusion complexes using the supercritical fluid technology involves the substitution of water molecules in the central cavity of CDs with drug molecules. The higher the temperature, the more effective the substitution process.

#### 2.4.3. Pressure of the Supercritical Fluid

By keeping several process parameters constant (i.e., the molar ratio of baicalin and HP-β-CD = 1:2; the complexation time = 8 h; the temperature of complexation chamber = 55 °C), the impact of the pressure of the supercritical fluid on the process of inclusion complexation was examined. Our results showed that the inclusion efficiency, drug loading efficiency, and water solubility of the generated complexes were the highest when the pressure of the supercritical fluid was at 20 MPa (Table 3). Setting the pressure too high or too low also lowered the inclusion efficiency and drug loading efficiency of the complexation process, and reduced the solubility of the product.

#### 2.4.4. Molar Ratio of Baicalin and HP-β-CD

By keeping several process parameters constant (i.e., the pressure of the supercritical fluid = 20 MPa; the complexation time = 8 h; the temperature of complexation chamber = 55 °C), the molar ratio of baicalin and HP-β-CD showed a close relationship with the inclusion efficiency and water solubility of the generated complexes (Table 4). In particular, the inclusion efficiency increased from 0.975% to 3.516% when the molar ratio increased from 1:1 to 1:4.

#### 2.4.5. Orthogonal Optimization of the Complexation Process

To select the conditions for effective complexation, orthogonal optimization was performed. Complexation time was set as 8 h in accordance to the result of our single-factor experiment (Table 1). The table of the orthogonal factor levels and the results of the orthogonal test are shown in Table 5 and Table 6, respectively. Based on the R values obtained, the degree of inference for each of the factors on the inclusion efficiency and drug loading efficiency was different. The order of the degree is C > A > B. As shown by the extreme values R, A_2_B_2_C_3_ is an optimal prescription, and presents excellent properties. Furthermore, the inclusion efficiency was 3.516%, and the drug loading efficiency was 0.227%. The solubility of this sample was over 1000 μg/mL, which is much higher than that of pure baicalin.

### 2.5. Addition of Organic Acids and Bases on Inclusion Complexation

Based on the results of our orthogonal experiments, lysine or citric acid was added to improve the inclusion efficiency and drug loading efficiency of the complexation process. By keeping several process parameters constant (i.e., the complexation pressure = 20 MPa; complexation time = 8 h; temperature of the complexation chamber = 55 °C; molar ratio of baicalin and HP-β-CD = 1:4), the effects of the addition of organic acids and bases on the inclusion efficiency and drug loading efficiency were examined. Our results showed that the addition of citric acid failed to improve the inclusion efficiency and drug loading efficiency, but both of the efficiency measures decreased (Table 7). In contrast, the addition of lysine (an organic base) could greatly increase the inclusion efficiency by more than 6-fold, which was related to the acidity of baicalin. The addition of an alkali can promote the inclusion of baicalin by HP-β-CD.

The amount of CD that can be used in most of the preparations is limited due to the cost, toxicity, and dose. Over the years, different methods have been proposed to improve the inclusion efficiency. Representative examples include the incorporation of auxiliary agents and amino acids during the inclusion complexation process [18,19,25]. In this study, lysine was used rather than supercritical ammonia as the latter is highly corrosive. Using lysine can prevent the damage caused to the high pressure stainless steel chamber. By adding organic acids or organic bases, the inclusion efficiency can be increased significantly. After adding an organic alkali, the enhancement of the inclusion efficiency was obvious, and this is consistent with a previous report [26].

## 3. Materials and Methods

### 3.1. Preparation of Inclusion Complexes by Solution Mixing

HP-β-CD-based inclusion complexes were fabricated using a solution agitation method as previously described [12]. In brief, 36.2 g of HP-β-CD was dissolved in 177.3 mL of water under magnetic stirring at 60 °C. Ten grams of baicalin was added slowly into the complexation chamber (molar ratio of baicalin and CD = 1:1). After continuous stirring at 60 °C for 2 h, the mixture was cooled to room temperature and stayed at ambient conditions for 2 h before it was filtered by a 0.45-μm filter membrane. After lyophilization, the final products were collected. The products were found to have an inclusion efficiency of 1.51%, the drug loading efficiency was 0.33%, and the solubility was 0.30 mg/mL; whereas the solubility of pure baicalin was found to be only 0.08 mg/mL.

### 3.2. Preparation of Inclusion Complexes by Supercritical Fluid Encapsulation

HP-β-CD and baicalin were first mixed in a complexation chamber at different temperatures (45 °C, 55 °C, and 65 °C) and at different molar ratios (1:2, 1:3, 1:4), followed by the addition of a CO_2_ supercritical fluid at different pressures (10 MPa, 20 MPa, and 30 MPa). The complexation mixture was kept at high pressure for different time periods (3, 6, 10, and 14 h) to generate the inclusion complexes. 

### 3.3. Drug Content Determination by HPLC

The chromatographic column used was a ZORBAX Eclipse XDB-C_18_ (250 mm × 4.6 mm, 5 μm) accommodated by Agilent Technologies, Santa Clara, CA, USA. The mobile phase was constituted by methanol, water, and phosphoric acid, with the component proportions being 47:53:0.2. The number of theoretical plates was not less than 2500 in the wavelength of 280 nm. The flow rate was 1 mL·min^−1^ and the sampling quantity was 10 μL. According to the drug content determined by HPLC, the loading efficiency and inclusion efficiency were calculated according to the following formulae.

(1) $$\mathrm{Inclusion}\;\mathrm{efficiency}\;\left(\%\right)=\frac{m_{L}}{m_{T}}\;\times 100\%$$

(2) $$\mathrm{Loading}\;\mathrm{efficiency}\;\left(\%\right)=\frac{m_{L}}{m_{I}}\;\times 100\%$$

where m_L_ is the mass of the drug loaded into the inclusion complex; m_I_ is the mass of the inclusion complexes; and m_T_ is the total mass of the drug added during the inclusion complexation process.

### 3.4. Characterization by Powder XRD

X-ray diffraction analysis was conducted by an Empyrean X-ray diffractometer (Malvern Panalytical, Inc., Heracles Almelo, The Netherlands), using Cu Kα radiation, operating at 40 KV and 40 mA in the angular range 3° < 2θ < 50° with an acquisition step of 0.02° and counting time of 40 s/step. The divergent slit was set as 1/32°. An anti-scattering slit of 1/16° and 7.5 mm was used.

### 3.5. Characterization by DSC

Samples were analyzed by a Netsch DSC 204F1 differential scanning calorimeter (Netsch, Inc., Serb, Germany). During the experiment, the samples were heated from room temperature to 300 °C at a heating rate of 10 °C/min under a nitrogen flow at 20 mL/min.

### 3.6. Characterization by Fourier-Transform IR Spectroscopy

The potassium bromide pellet method was adopted to detect the samples by infrared spectroscopy (IR) (FTIR 8400 spectrometer, Shimadzu, Inc., Kyoto, Japan). Briefly, 1.0 mg samples and an appropriate amount of KBr were added into the mortar to be ground uniformly. Then, the pellet mixture was scanned at a wavelength range of 4000–400 cm^−1^.

### 3.7. Characterization by SEM

Powder samples were dispersed on an aluminum stub with a thin self-adhered carbon film, and were coated with a thin layer of gold using an ion sputter under an argon atmosphere. The surface morphology of the sample was observed using a scanning electron microscope (Quanta400F, FEI, Inc., Hillsboro, OR, USA).

### 3.8. Particle Size Analysis

The average diameter of baicalin, HP-β-CD, and the inclusion complexes were detected by a laser particle size analyzer (Mastersizer 3000, Malvern Panalytical, Inc., Heracles Almelo, The Netherlands). The instrument enabled the measurement of the particle size in a range between 0.01 and 3500.0 μm. The method used for analysis was the dry method.

### 3.9. Solubility Determination

An excessive amount of baicalin (or the inclusion complexes) was added into 5 mL of distilled water to obtain a supersaturated solution. The solution was agitated continuously in a water bath at 25 °C. Samples were taken after 24 and 48 h, and were filtered through a 0.45-μm filter (Selby-Biolab HPLC-certified, Mulgrave, VIC, Australia) prior to analysis. The drug content of the samples was determined by HPLC, and the solubility was calculated as an average of three measurements.

### 3.10. Orthogonal Optimization of the Process

The temperature of the complexation chamber (A), pressure of the supercritical fluid (B), and the molar ratio (baicalin:HP-β-CD) (C) were used as the three process parameters to be studied in the orthogonal test. The L9 (3^4^) orthogonal table was formulated. D is the column where the deviation values are listed. Taking the inclusion efficiency and drug loading efficiency as the evaluation indices (V), the comprehensive score method was applied and the weight coefficients of the two efficiencies were 0.7 and 0.3, respectively. The results were statistically analyzed by intuitive analysis to determine the degree of the effects of different factors on the complexation process. 

## 4. Conclusions

To demonstrate the efficiency of enhancing the solubility of hydrophobic drugs using CD-based inclusion complexation mediated by supercritical fluid encapsulation, in this study, baicalin was used as a model drug. Compared to those generated by conventional solution mixing, the complexes prepared by our method showed more favorable properties (e.g., narrower particle size distribution, and smaller particle size). Our results suggest that CD-based inclusion complexation mediated by the supercritical fluid technology may help to solve the problems encountered by the existing preparation methods. Along with its capacity of generating inclusion complexes in one-step, our method has the potential to expand the application potential of water-insoluble drugs, widening their use in pharmaceutical formulation and treatment development.

## Figures and Tables

**Table 1:** Effects of the length of complexation time on the inclusion efficiency, drug loading efficiency, and water solubility of the generated complexes.

Complexation Time (h)	Inclusion Efficiency (%)	Loading Efficiency (%)	Water Solubility (μg/mL)
3	1.477	0.179	633.26
6	1.482	0.180	787.36
10	1.536	0.186	856.84
14	1.364	0.166	1183.76

**Table 2:** Effects of the complexation temperature on the inclusion efficiency, drug loading efficiency, and water solubility of the generated complexes.

Temperature (°C)	Inclusion Efficiency (%)	Loading Efficiency (%)	Water Solubility (μg/mL)
45	1.769	0.214	1106.66
55	1.909	0.232	1207.17
65	1.544	0.187	1117.43

**Table 3:** Effects of the supercritical fluid pressure on the inclusion efficiency, drug loading efficiency, and water solubility of the generated complexes.

Pressure (MPa)	Inclusion Efficiency (%)	Loading Efficiency (%)	Water Solubility (μg/mL)
10	1.327	0.161	1274.62
20	1.909	0.232	1207.17
30	1.377	0.167	1281.47

**Table 4:** Effects of the molar ratio (baicalin:HP-β-CD) on the inclusion efficiency, drug loading efficiency, and water solubility of the generated complexes.

Molar ratio (Baicalin:HP-β-CD)	Inclusion Efficiency (%)	Loading Efficiency (%)	Water Solubility (μg/mL)
1:1	0.975	0.211	1232.31
1:2	1.909	0.232	1207.17
1:3	2.328	0.196	1179.50
1:4	3.516	0.227	1096.01

**Table 5:** Table of the orthogonal factor levels.

Level	Factors		
	A	B	C
	Temperature of the Complexation Chamber (°C)	Pressure of the Supercritical Fluid (MPa)	Molar Ratio (Baicalin:HP-β-CD)
1	50	15	1:2
2	55	20	1:3
3	60	25	1:4

**Table 6:** Results of the orthogonal test.

No.	Factors				Index		Comprehensive Index
	A	B	C	D	A	B	V
	Temperature of the Complexation Chamber (°C)	Pressure of the Supercritical Fluid (MPa)	Molar Ratio (baicalin:HP-β-CD)	Deviation	Loading Efficiency (%)	Inclusion Efficiency (%)	
1	1	1	1	1	0.1426	1.1754	0.8656
2	1	2	2	2	0.1477	1.7527	1.2712
3	1	3	3	3	0.1611	2.4907	1.7918
4	2	1	2	3	0.1438	1.7144	1.2432
5	2	2	3	1	0.1682	2.6069	1.8753
6	2	3	1	2	0.1546	1.2737	0.9380
7	3	1	3	2	0.1515	2.3537	1.6930
8	3	2	1	3	0.1490	1.2276	0.9040
9	3	3	2	1	0.1256	1.4886	1.0797
Ij	1.31	1.27	0.90	1.27			
IIj	1.35	1.35	1.20	1.30			
IIIj	1.23	1.27	1.79	1.31			
R	0.13	0.08	0.88	0.04			

**Table 7:** Effects of the addition of organic acids and bases on the inclusion efficiency and drug loading efficiency.

	Inclusion Efficiency (%)	Loading Efficiency (%)
Lysine	66.48	4.27
Citric acid	0.97	0.06
